# Exploring humanistic burden of fatigue in adults with multiple sclerosis: an analysis of US National Health and Wellness Survey data

**DOI:** 10.1186/s12883-023-03423-z

**Published:** 2024-02-01

**Authors:** Hoa H. Le, Jennifer Ken-Opurum, Anne LaPrade, Martine C. Maculaitis, John J. Sheehan

**Affiliations:** 1 Janssen Scientific Affairs, LLC, Titusville, NJ USA; 2 Cerner Enviza, North Kansas City, MO USA

**Keywords:** Multiple Sclerosis, Relapse-Remitting Multiple Sclerosis, Fatigue, Quality of Life, Mental Health

## Abstract

**Background:**

This retrospective study examined the humanistic burden of fatigue in patients with relapsing-remitting multiple sclerosis (RRMS), compared with adults without MS, using data from the 2017 and 2019 US National Health and Wellness Survey.

**Methods:**

The 5-item Modified Fatigue Impact Scale (MFIS-5) was used to assess level of fatigue (MFIS-5 score <15: low fatigue [LF]; MFIS-5 score ≥15: high fatigue [HF]) in patients with RRMS. Health-related quality of life (HRQoL) measures (Short Form 36-Item Health Survey version 2, Euroqol-5 Dimensions-5 Levels [EQ-5D-5L], Patient Health Questionnaire-9 [PHQ-9], Generalized Anxiety Disorder-7 [GAD-7], Perceived Deficits Questionnaire-5) and treatment-related characteristics were assessed.

**Results:**

In total, 498 respondents were identified as RRMS (*n=*375 RRMS+LF, *n=*123 RRMS+HF) and compared with 1,494 matched non-MS controls. RRMS+LF and RRMS+HF had significantly lower Short Form 6 Dimensions health utility, Mental and Physical Component Summary, and EQ-5D-5L scores and higher PHQ-9 and GAD-7 scores, compared with matched non-MS controls (all *p<*0.001); scores were worse for RRMS+HF than RRMS+LF across all measures (all *p<*0.001). A higher proportion of RRMS+HF reported moderate-to-severe depression and moderate-to-severe anxiety, compared with RRMS+LF and matched non-MS controls (both *p<*0.001). Fatigue was a significant predictor of poor HRQoL across all measures (all *p<*0.001).

**Conclusions:**

Patients with RRMS experienced lower HRQoL with higher levels of fatigue, highlighting an unmet need. Results may help to inform physician-patient communication and shared decision-making to address fatigue and its associated impact on patients’ HRQoL.

## Background

Multiple sclerosis (MS), a chronic inflammatory and autoimmune disorder of the central nervous system, is characterized by demyelination and axonal loss [1]. In most cases, patients with MS are diagnosed during their productive years of life, with diagnosis peaking between ages 20 to 40 years [2]. Based on the extrapolation of health claims data, the 2017 prevalence of MS in the United States (US) was estimated to be about 1 million individuals [3]. Course of disease and progression are highly unpredictable and characterized by relapses and periods of remission. Relapsing-remitting MS (RRMS), the most prevalent form of MS, is diagnosed in approximately 85% of patients with MS [4]. RRMS is characterized by temporary exacerbations of neurological symptoms resulting in progressive neurological decline with either partial or no recovery [4, 5].

As a result of demyelination or axonal loss, patients with MS report a broad range of symptoms, including muscle stiffness, restricted mobility, fatigue, and pain, resulting in poorer health-related quality of life (HRQoL) [6–8]. For example, symptoms including fatigue, difficulty balancing/walking, numbness, difficulty remembering, pain, and muscle spasms were associated with lower HRQoL in patients with RRMS [8]. Fatigue, one of the most common symptoms of MS, is experienced by about 80% of patients with MS, of whom 55% reported it as one of the worst symptoms experienced, regardless of the level of disability [9]. As per the North American Research Committee on MS Registry (*N=*35,000), fatigue was reported by about 80% of patients within the first year after onset [10]. Fatigue experienced by patients with MS can be more severe than fatigue among the general population, resulting in sudden episodes on a daily basis that may worsen as the day progresses and can be aggravated by heat and humidity [11]. Numerous other studies have reported that patients with MS with fatigue have poor HRQoL [12–14]. For example, fatigue was shown to be positively correlated with disability and negatively correlated with HRQoL in patients with MS [12]. Additionally, higher levels of fatigue were associated with a greater risk for developing depression [15] and poor physical and mental health [14]. Furthermore, fatigue was also reported as a likely predictor of disease progression [16].

A number of studies have shown that MS imposes a significant humanistic and economic burden to patients and the healthcare system [17–21]. Analysis of the US National Health and Wellness Survey (NHWS) indicated that patients with MS had significant work-related impairment and greater healthcare resource utilization (HCRU), compared with the non-MS control group [17]. A retrospective cross-sectional analysis of US NHWS data among employed patients with RRMS also reported poorer HRQoL, lower work productivity, and higher HCRU, compared with those without MS [18]. Additionally, a more recent analysis of US NHWS data showed that patients with RRMS and higher fatigue status disproportionally experienced greater economic burden and reduced work productivity than patients with lower fatigue or those without MS [21].

Previous research has investigated the effect of MS-related symptoms on patient-reported outcomes [8], work productivity [8, 18], HCRU [18], and overall impact on HRQoL [12, 13, 18, 22]. These studies identified fatigue as one of the predictors for poor HRQoL, yet the effects of fatigue on the physical and mental components of HRQoL are not well understood [8, 12, 13, 18, 22]. Although Tabrizi and Radfar [14] reported an incremental effect of fatigue level on HRQoL, to our knowledge, no existing research has specifically examined the influence of level of fatigue on overall HRQoL in patients with RRMS compared with the general population. Exploring this impact is necessary to identify and address unmet needs such as early diagnosis of fatigue, improved treatment, and monitoring. This retrospective, exploratory study was designed to examine the overall humanistic burden of fatigue in terms of HRQoL and mental and physical health in patients with RRMS, compared with adults without MS.

## Methods

### Study design

**Fig. 1:**
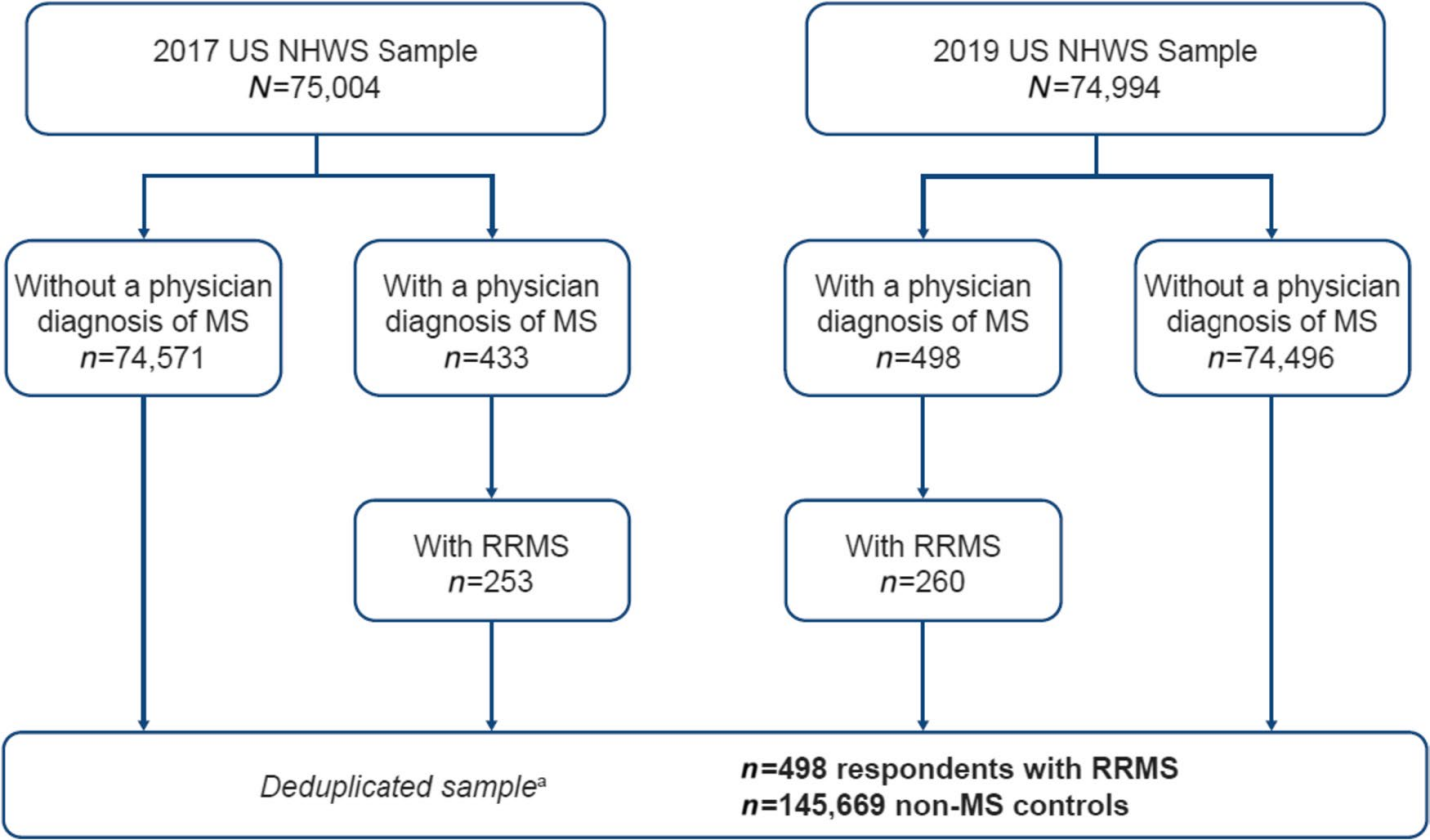
Study Sample Flow Chart. Note: MS, Multiple Sclerosis; NHWS, National Health and Wellness Survey; RRMS, Relapsing-Remitting Multiple Sclerosis; US, United States. ^a^For respondents taking the NHWS in both 2017 and 2019, only their most recent data were used

This retrospective observational study was conducted using data from the US NHWS, a nationally representative, cross-sectional, self-administered, internet-based survey that collects data annually from approximately 75,000 respondents. The present study used data from the 2017 (*n=*75,004) and 2019 (*n=*74,994) surveys (Fig. 1).

NHWS respondents are recruited through a web-based consumer panel via opt-in e-mails, co-registration with panel partners, e-newsletter campaigns, banner placements, and affiliate networks. Respondents explicitly agree to be a panel member, register with the panel through a unique e-mail address, and complete an in-depth demographic registration profile. For respondents completing the survey in both years, only their most recent data were included in analysis. A quota sampling procedure, with strata by sex, race/ethnicity, and age, is implemented to ensure that the demographic composition of the NHWS sample is representative of the general adult population in the US.

#### Data availability

NHWS is a proprietary database owned by Cerner Enviza, and therefore the datasets analyzed in this study are not publicly available.

### Study population

The RRMS study sample included respondents aged ≥18 years who resided in the US, reported being diagnosed with MS by a healthcare provider, and reported having RRMS. The non-MS (control) population included respondents aged ≥18 years who resided in the US and did not report MS diagnosed by a healthcare provider.

#### Ethics

All research methods were carried out in accordance with the Declaration of Helsinki. Experimental protocols for the NHWS were reviewed by Pearl Institutional Review Board (Indianapolis, IN) and granted exemption status. Informed consent was electronically obtained from US NHWS respondents.

### Study measures

#### Sociodemographic and health characteristics

Sociodemographic characteristics, including age, sex, race, marital status, education, employment status, annual household income, and insurance type, were analyzed. Health characteristics including Charlson comorbidity index (CCI) score [23], body mass index, smoking status, alcohol consumption, and frequency of exercise in the past month were also analyzed.

#### MS-specific characteristics

Characteristics of MS treatment, including current medications used, satisfaction with current medications, and reasons for switching from previous to current medications, were analyzed.

#### Fatigue

The 5-item Modified Fatigue Impact Scale (MFIS-5), an abbreviated version of the 21-item MFIS, assesses the burden of MS-related fatigue on cognitive, physical, and psychosocial functioning based on responses provided by patients to 5 questions [24, 25]. On each item, the impact of fatigue on patients’ activities was rated on a 5-point Likert scale (0 = never, 1 = rarely, 2 = sometimes, 3 = often, 4 = almost always); responses are then summed across all items so that the total score ranges from 0 to 20. Higher total scores indicate greater burden from MS-related fatigue.

#### Humanistic burden: HRQoL

##### *Medical Outcomes Study 36-Item Short Form Health Survey (version 2)*

The Mental Component Summary (MCS) and Physical Component Summary (PCS) scores from the 36-Item Short Form Health Survey (version 2; SF-36v2; https://www.rand.org/health-care/surveys_tools/mos/36-item-short-form/survey-instrument.html) were used to assess mental (vitality, role functioning, mental health, and social functioning) and physical (general health perceptions, physical functioning, bodily pain, and role functioning) health status, respectively. Scores on the MCS and PCS range from 0 to 100, with higher scores indicating better health status [26]. The Short Form-6 Dimension (SF-6D) health utility score, derived from the SF-36v2, provides a preference-based index score ranging from 0 to 1, with higher scores indicating better overall general health [27].

##### Euroqol-5 Dimensions-5 Levels (EQ-5D-5L)

The EQ-5D-5L consists of a descriptive system and a visual analogue scale (EQ-5D-5L VAS). The EQ-5D-5L descriptive system includes 5 dimensions: mobility, self-care, usual activities, pain/discomfort, and anxiety/depression. Each dimension has levels indicative of the extent of problems; an index score encompassing all 5 dimensions is derived, in which 0 indicates a health state equivalent to death and 1 indicates a health state equivalent to perfect health [28]. The VAS item allows respondents to indicate their self-rated health with the endpoints on the line being “Best imaginable health state” and “Worst imaginable health state.”

##### *Patient *health* questionnaire-9*

The Patient Health Questionnaire-9 (PHQ-9) was used to measure the severity of depression. The PHQ-9 evaluates the frequency in the past 2 weeks of 9 *Diagnostic and Statistical Manual of Mental Disorders, Fourth Edition* depression symptoms. Scores on the PHQ-9 range from 0 to 27, with higher scores indicating greater severity of depression [29]. Scores of 5, 10, 15, and 20 represent cutoffs for mild, moderate, moderately severe, and severe depression, respectively.

##### Generalized anxiety disorder-7

Anxiety symptom severity was measured using the Generalized Anxiety Disorder-7 (GAD-7). The GAD-7 (https://adaa.org/sites/default/files/GAD-7_Anxiety-updated_0.pdf) is a 7-item general anxiety measure indicating the effect of different anxiety symptoms on the respondent over the prior 2 weeks. Each item was scored from 0 to 3, providing a total score of 0 to 21. Scores of 5, 10, and 15 are the cutoffs for mild, moderate, and severe anxiety, respectively [30].

#### MS -specific outcomes

##### *Perceived *deficits* questionnaire-5*

MS-related cognitive dysfunction was measured using the Perceived Deficits Questionnaire-5 (PDQ-5). The PDQ-5 (https://howdenmedicalclinic.com/wp-content/uploads/2018/04/PDQ-D5.pdf) assesses the impact of MS on specific cognitive domains, such as attention, retrospective memory, prospective memory, and planning/organization [31]. Respondents reported the frequency that each cognitive symptom was experienced on a scale from 0 (never) to 4 (almost always). Responses to all 5 questions were summed, and a composite score (ranging from 0-20) was computed, with higher scores indicating greater cognitive impairment.

### Statistical analysis

Propensity score matching was conducted to minimize baseline differences in demographic and health characteristics between patients with RRMS and non-MS controls using binary logistic regression. Age, sex, race, and CCI score were selected *a priori* as covariates for matching. Respondents with RRMS were matched to non-MS controls (ratio: 1:3) using a greedy-matching algorithm from the R (R Foundation for Statistical Computing; Vienna, Austria) MatchIt package [32]. Following the matching procedure, a bivariate analysis was conducted on demographic and health characteristics to ensure matched sample groups.

Patients with RRMS were categorized by low (total MFIS-5 score <15) versus high (total MFIS-5 score ≥15) fatigue (ie, RRMS+low fatigue [LF] vs RRMS+high fatigue [HF]) [33, 34].

As this study was exploratory and not designed to conform to a set of pre specified hypotheses, the study results are reported without adjustment for multiple testing. However, when multiple tests were performed, a false discovery rate approach was used to evaluate the results after correcting for family-wise error [35]. The pre specified false discovery (i.e., Type 1 error) rate used to calculate the critical values for each set of statistical tests was 0.05. Compared to a Bonferroni correction, the false discovery rate approach is a more powerful procedure and is less sensitive to increases in the number of tests [36].

#### Descriptive statistics

Sociodemographic and health characteristics of all 3 groups (RRMS+LF, RRMS+HF, and non-MS controls) were reported using descriptive statistics: continuous or discrete variables were reported using means and standard deviations, and categorical variables were reported using frequencies and percentages.

#### Bivariate analysis

Independent-samples *t*-test or 1-way analysis of variance tests were conducted for continuous variables, with Chi-square tests for categorical variables. *P* values <0.05 (2-tailed) were considered statistically significant.

#### Multivariable analysis

Generalized linear models (GLMs) specifying normal distribution and identity-link function were used for normally distributed outcome variables (i.e., MCS, PCS, SF-6D health utility, EQ-5D-5L index, EQ-5D-5L VAS, treatment satisfaction, and PDQ-5 scores). GLMs specifying negative binomial distribution and log-link function were used for skewed outcome variables (i.e., PHQ-9 and GAD-7 scores). Binary logistic regression analyses were used to assess the association between level of fatigue (RRMS+LF vs RRMS+HF) and fatigue (as continuous variable) with moderate-to-severe depression symptoms (PHQ-9 score ≥10) and moderate-to-severe anxiety symptoms (GAD-7 score ≥10). All outcomes in multivariable analyses were modeled separately, with age, sex, race, and CCI score as covariates. *P* values <0.05 (2-tailed) were considered statistically significant.

## Results

### Propensity score matching results

Prior to matching, the general population sample had a mean age of 47.22±17.43 years, 54.67% were women, 74.37% were white, and mean CCI score was 0.44±1.04 (data not shown). Post matching, these variables did not differ between the non-MS controls and RRMS respondents (mean age: 50.98±13.08 vs 50.52±12.80 years; 75.44% vs 74.70% women; 81.46% vs 81.53% white; mean CCI score, 0.68±1.80 vs 0.79±2.63; all *p>*0.05) (Table 1).

**Table 1:** Sample sociodemographic and health characteristics

		**Matched** **Non-MS Controls**		**RRMS**		***p* Value**	**RRMS+LF** **(MFIS-5 = 0-14)**		**RRMS+HF** **(MFIS-5 = 15+)**		***p* Value**
		**(*N=*1,494)**		**(*N=*498)**			**(*N=*375)**		**(*N=*123)**		
		**Mean/*n***	**SD/%**	**Mean/*n***	**SD/%**		**Mean/*n***	**SD/%**	**Mean/*n***	**SD/%**	
**Age (years)**		50.98	13.08	50.52	12.80	0.500	51.12	12.80	48.71	12.67	0.070
**Sex**	Male	367	24.56	126	25.30	0.787	94	25.07	32	26.02	0.928
	Female	1,127	75.44	372	74.70		281	74.93	91	73.98	
**Race**	White	1,217	81.46	406	81.53	0.999	301	80.27	105	85.37	0.385
	Black/African American	221	14.79	74	14.86		59	15.73	15	12.20	
	Asian	18	1.20	6	1.20		4	1.07	2	1.63	
	Some other race or origin	38	2.54	12	2.41		11	2.93	1	0.81	
**Marital Status (% yes)**	Married/living with a partner	911	60.98	312	62.65	0.686	242	64.53	70	56.91	0.129
**Education (% yes)**	University degree	698	46.72	222	44.58	0.436	169	45.07	53	43.09	0.781
**Employment Status**	Employed full time	670	44.85	137	27.51	<0.001	115	30.67	22	17.89	<0.001
	Self-employed	95	6.36	16	3.21		12	3.20	4	3.25	
	Employed part time	126	8.43	28	5.62		23	6.13	5	4.07	
	Homemaker	120	8.03	54	10.84		43	11.47	11	8.94	
	Retired	279	18.67	124	24.90		101	26.93	23	18.70	
	Student	19	1.27	7	1.41		4	1.07	3	2.44	
	Long-term disability	91	6.09	100	20.08		53	14.13	47	38.21	
	Not employed, but looking for work	71	4.75	19	3.82		15	4.00	4	3.25	
	Not employed and not looking for work	23	1.54	13	2.61		9	2.40	4	3.25	
**Annual Household Income**	<$50,000	564	37.75	241	48.39	<0.001	164	43.73	77	62.60	0.001
	$50,000 to $74,999	284	19.01	82	16.47		63	16.80	19	15.45	
	≥$75,000	582	38.96	160	32.13		134	35.73	26	21.14	
	Decline to answer	64	4.28	15	3.01		14	3.73	1	0.81	
**Insurance Type**^**a**^	Commercially insured	928	64.04	239	49.08	<0.001	190	51.77	49	40.83	0.090
	Medicaid	132	9.11	43	8.83		29	7.90	14	11.67	
	Medicare	281	19.39	181	37.17		129	35.15	52	43.33	
	Other type of insurance (VA/CHAMPUS, TRICARE, not sure)	45	3.11	17	3.49		15	4.09	2	1.67	
	Not insured	63	4.35	7	1.44		4	1.09	3	2.50	
**Charlson Comorbidity Index Score**		0.68	1.80	0.79	2.63	0.304	0.66	2.85	1.16	1.79	0.067
**Body Mass Index (kg/m**^**2**^**)**		29.27	7.50	28.97	7.79	0.452	28.76	7.41	29.63	8.86	0.288
**Smoking Status (% yes)**	Current smoker	254	17.00	101	20.28	0.098	68	18.13	33	26.83	0.037
**Alcohol Use (% yes)**	Drinks alcohol	1006	67.34	311	62.45	0.052	238	63.47	73	59.35	0.477
**Days Exercising in Past Month**		7.71	8.80	6.7	8.95	0.027	7.41	9.24	4.54	7.64	0.002
**MFIS-5 Score among RRMS**		NA	NA	10.56	4.97	NA	8.50	3.84	16.82	1.71	<0.001

For the set of tests shown in the table, the corrected alpha levels were 0.012 and 0.014 for comparisons between RRMS and controls and between RRMS+LF and RRMS+HF, respectively. The differences between RRMS+LF and RRMS+HF in smoking status (*p=*0.037 > *p=*0.014) and differences between RRMS and controls in days exercised in the past month (*p=*0.027 > *p=*0.012) were no longer statistically significant

*CHAMPUS* Civilian Health and Medical Program of the Uniformed Services, *HF* high fatigue, *LF* low fatigue, *MFIS-5* 5-item Modified Fatigue Impact Scale, *MS* multiple sclerosis, *NA* not applicable, *RRMS* relapsing-remitting multiple sclerosis, *SD* standard deviation, *VA* Veterans Affairs

^a^Percent values were calculated using non missing data: non-MS matched controls, *n=*1,449; RRMS, *n=*487; RRMS+LF, *n=*367; RRMS+HF, *n=*120

### RRMS sample characteristics

Among the 498 respondents reporting RRMS, most were married or living with a partner (62.65%). Approximately one-third were employed (36.34%), and 48.39% had an annual household income of <$50,000. Mean MFIS-5 score was 10.56±4.97 (Table 1).

The majority of sociodemographic and health characteristics were similar between the RRMS+LF (*n=*375) and RRMS+HF (*n=*123) groups (Table 1). However, statistically significant differences were observed for employment status (*p<*0.001), with RRMS+HF less likely to be employed full-time (17.89% vs 30.67%) and more likely to be on long-term disability (38.21% vs 14.13%), compared with RRMS+LF. Furthermore, a significantly higher proportion of RRMS+LF reported their annual income as >$75,000 than RRMS+HF (35.73% vs 21.14%; *p=*0.001). Mean MFIS-5 score was 8.50±3.84 for RRMS+LF and 16.82±1.71 for RRMS+HF (*p<*0.001) (Table 1).

There were no differences between the RRMS+LF and RRMS+HF groups in the percentages of respondents currently using a prescription for the treatment of MS or having previously been using a different prescription (Table 2).

**Table 2:** MS prescription use, treatment satisfaction, and reasons for switching medications

	**RRMS+LF (MFIS-5=0-14)**		**RRMS+HF (MFIS-5=15+)**		***p* Value**
	**(*N=*375)**		**(*N=*123)**		
	**Mean/*n***	**SD/%**	**Mean/*n***	**SD/%**	
**MS Prescription Use (% yes)**					
Current Use	278	74.13	88	71.54	0.655
Prior Use	157	98.12	47	94.00	0.297
**Overall Treatment Satisfaction**^**a**^	5.73	1.20	4.89	1.58	<0.001
**Reasons for Switching MS Medication (% yes)**					
Physician Recommendation	89	56.69	18	38.30	0.041
Side Effects	43	27.39	22	46.81	0.020
Lower Cost	4	2.55	2	4.26	0.908
Not Effective	48	30.57	15	31.91	>0.999
Dosing	24	15.29	7	14.89	>0.999
Mode of Administration	39	24.84	12	25.53	>0.999
Other	12	7.64	3	6.38	>0.999

For the set of tests shown in the table, the corrected alpha level was 0.005 for comparisons between RRMS+LF and RRMS+HF. The differences between RRMS+LF and RRMS+HF in reporting side effects (*p=*0.020 > *p=*0.005) and physician recommendation (*p=*0.041 > *p=*0.005) as reasons for switching MS medication were no longer statistically significant

*HF* high fatigue, *LF* low fatigue, *MFIS-5* 5-item Modified Fatigue Impact Scale, *MS* multiple sclerosis, *RRMS* relapsing-remitting multiple sclerosis, *SD* standard deviation

^a^On a 7-point Likert scale in which 1 = extremely dissatisfied and 7 = extremely satisfied

Overall treatment satisfaction was significantly higher in the RRMS+LF group than RRMS+HF group (*p<*0.001) (Table 2). RRMS+HF were significantly less likely to switch their MS medication due to physician recommendation (38.30% vs 56.69%; *p=*0.041), but more likely to switch due to side-effects (46.81% vs 27.39%; *p=*0.020), compared with RRMS+LF (Table 2).

### HRQoL comparisons between matched non-MS controls, RRMS+LF, and RRMS+HF

**Fig. 2:**
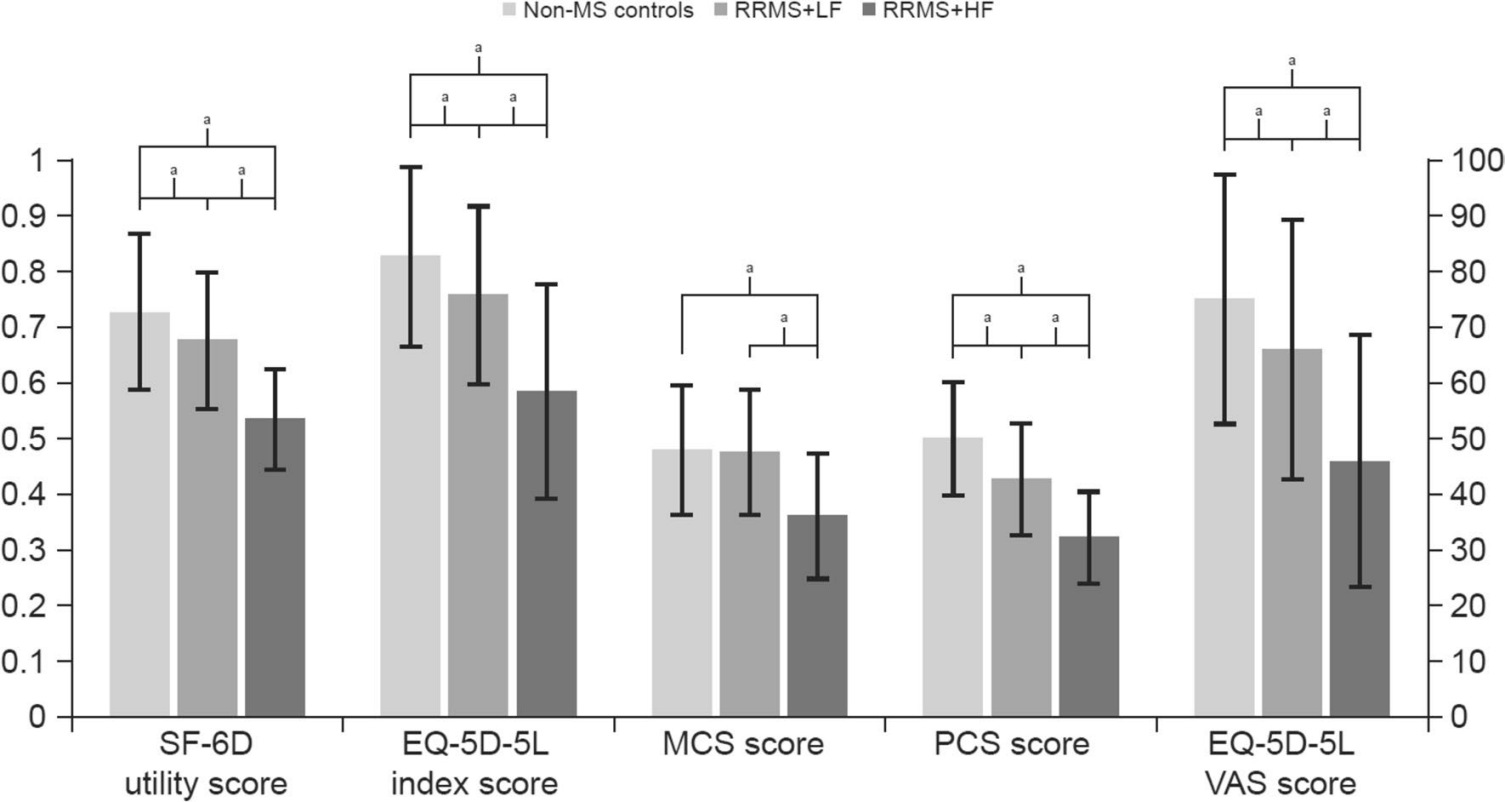
SF-36v2 and EQ-5D-5L, Comparisons Between Matched Non-MS Controls and RRMS+LF and RRMS+HF. Note: For the set of tests shown in the figure, the corrected alpha level was 0.050 for the omnibus test and for comparisons between RRMS+LF and RRMS+HF and between RRMS+HF and controls; the corrected alpha level was 0.028 for comparisons between RRMS+LF and controls. Differences remained statistically significant after adjusting the alpha level. EQ-5D-5L, Euroqol-5 Dimensions-5 Levels; HF, high fatigue; LF, low fatigue; MS, multiple sclerosis; MCS, Mental Component Summary; PCS, Physical Component Summary; RRMS, relapsing-remitting multiple sclerosis; SF-36v2, 36-Item Short Form Health Survey (version 2); SF-6D, Short Form-6 Dimensions; VAS, visual analogue scale. SF-6D utility and EQ-5D-5L index are on a scale of 0-1.0; MCS, PCS, and EQ-5D-5L VAS are on a scale of 0-100. ^a^*p<*0.001

Compared with matched non-MS controls, respondents in both RRMS+LF and RRMS+HF groups had significantly lower PCS, SF-6D health utility, EQ-5D-5L index, and EQ-5D-5L VAS scores (all *p<*0.001), while RRMS+HF also had lower MCS scores and higher PHQ-9 and GAD-7 scores (all *p<*0.001), indicating poorer HRQoL related to RRMS (Table 3; Fig. 2).

**Table 3:** Health-related quality of life, comparisons between matched non-MS controls and RRMS+LF and RRMS+HF

	**A. Matched** **Non-MS Controls**		**B. RRMS+LF** **(MFIS-5=0-14)**		**C. RRMS+HF** **(MFIS-5=15+)**		***p* Value**			
	**(*N=*1,494)**		**(*N=*375)**		**(*N=*123)**					
	**Mean/*n***	**SD/%**	**Mean/*n***	**SD/%**	**Mean/*n***	**SD/%**	**Omnibus**	**A vs B**	**A vs C**	**B vs C**
**SF-36v2**										
MCS Score	47.50	11.52	47.09	11.25	35.81	11.04	<0.001	0.539	<0.001	<0.001
PCS Score	49.56	10.09	42.31	9.81	31.97	8.08	<0.001	<0.001	<0.001	<0.001
SF-6D Utility Score	0.72	0.14	0.67	0.12	0.53	0.09	<0.001	<0.001	<0.001	<0.001
**EQ-5D-5L**										
EQ-5D-5L Index Score	0.82	0.16	0.75	0.16	0.58	0.19	<0.001	<0.001	<0.001	<0.001
EQ-5D-5L VAS Score	74.32	22.26	65.30	22.90	45.54	22.41	<0.001	<0.001	<0.001	<0.001
**PHQ-9 Score**	4.85	6.17	5.30	5.14	12.64	6.77	<0.001	0.200	<0.001	<0.001
**Depression Severity (PHQ-9)**										
None/minimal (0-4)	952	63.72	201	53.60	16	13.01	<0.001	<0.001	<0.001	<0.001
Mild (5-9)	281	18.81	108	28.80	24	19.51				
Moderate (10-14)	122	8.17	39	10.40	32	26.02				
Moderately Severe (15-19)	76	5.09	21	5.60	27	21.95				
Severe (20-27)	63	4.22	6	1.60	24	19.51				
**GAD-7 Score**	3.69	4.95	3.36	4.29	7.94	6.45	<0.001	0.232	<0.001	<0.001
**Anxiety Severity (GAD-7)**										
None/minimal (0-4)	1,037	69.41	263	70.13	46	37.40	<0.001	0.125	<0.001	<0.001
Mild (5-9)	249	16.67	75	20.00	29	23.58				
Moderate (10-14)	131	8.77	24	6.40	23	18.70				
Severe (15-21)	77	5.15	13	3.47	25	20.33				

For the set of tests shown in the table, the corrected alpha level was 0.050 for the omnibus test and for comparisons between RRMS+LF and RRMS+HF and between RRMS+HF and controls; the corrected alpha level was 0.028 for comparisons between RRMS+LF and controls. Differences remained statistically significant after adjusting the alpha level. *EQ-5D-5L *Euroqol-5 dimensions-5 levels, *GAD-7* Generalized anxiety disorder-7, *HF* High fatigue, *LF* Low fatigue, *MS* Multiple sclerosis, *MCS* Mental component summary, *PCS *Physical component summary, *PHQ-9* Patient health questionnaire-9, *RRMS* Relapsing remitting multiple sclerosis, *SD* Standard deviation, *SF-36v2* 36-Item short form health survey (version 2), *SF-6D* Short form-6 dimensions, *VAS* Visual analogue scale

Relative to RRMS+LF, respondents in the RRMS+HF group had significantly lower MCS, PCS, SF-6D health utility, EQ-5D-5L index, and EQ-5D-5L VAS scores and significantly higher PHQ-9 and GAD-7 scores, indicating poorer HRQoL related to fatigue in RRMS (all *p<*0.001) (Table 3; Fig. 2).

**Fig. 3:**
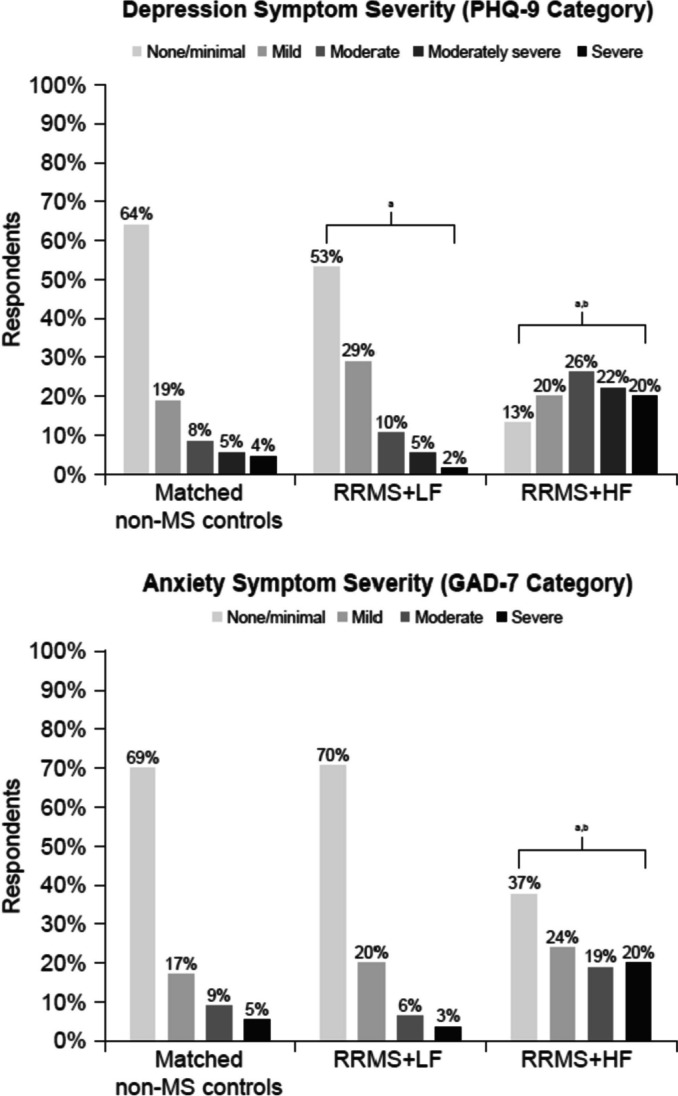
Depression and Anxiety Symptom Severity, Comparison Between Matched Non-MS Controls and RRMS+LF and RRMS+HF. Note: For the set of tests shown in the table, the corrected α level was 0.050 for the omnibus test and for comparisons between RRMS+LF and RRMS+HF and between RRMS+HF and controls; the corrected alpha level was 0.028 for comparisons between RRMS+LF and controls. Differences remained statistically significant after adjusting the alpha level. GAD-7, Generalized Anxiety Disorder-7; HF, high fatigue; LF, low fatigue; MS, multiple sclerosis; PHQ-9, Patient Health Questionnaire-9; RRMS, relapsing-remitting multiple sclerosis. PHQ-9 score cutoffs of 0-4 (mild/minimal), 5-9 (mild), 10-14 (moderate), 15-19 (moderately severe), and 20-27 (severe); GAD-7 score cutoffs of 0-4 (mild/minimal), 5-9 (mild), 10-14 (moderate), 15-21 (severe). ^a^*p<*0.001 for comparison to matched non-MS controls; ^b^*p<*0.001 for comparison to RRMS+LF

The distribution of depression symptom severity was significantly different between matched non-MS controls and RRMS+LF and RRMS+HF groups, as well as between RRMS+LF and RRMS+HF groups (Table 3; Fig. 3).

A higher percentage of respondents in the matched non-MS group had none/minimal depression, compared with the RRMS+LF and RRMS+HF cohorts (63.72% vs 53.06% vs 13.01%, respectively; *p<*0.001). A higher percentage of RRMS+HF had moderate-to-severe depression, compared with matched non-MS controls and RRMS+LF (67.48% vs 17.48% vs 17.06%, respectively; *p<*0.001).

The distribution of anxiety symptom severity was also significantly different between matched non-MS controls and RRMS+HF, as well as between RRMS+LF and RRMS+HF (Table 3; Fig. 3). A higher percentage of respondents in the matched non-MS and RRMS+LF groups reported none/minimal anxiety (69.41% and 70.13%, respectively), compared with the RRMS+HF group (37.40%; both *p<*0.001). A higher percentage of RRMS+HF had moderate-to-severe anxiety (39.03%), compared with matched non-MS controls and RRMS+LF (13.92% and 9.87%, respectively; both *p<*0.001).

### Humanistic outcomes related to fatigue in RRMS, multivariable results

In multivariable models, fatigue score was significantly and negatively associated with MCS, PCS, SF-6D health utility, EQ-5D-5L index, and EQ-5D-5L VAS scores, as well as overall treatment satisfaction, and significantly and positively associated with PHQ-9, GAD-7, and PDQ-5 scores (all *p<*0.01), suggesting poorer HRQoL is associated with a higher level of fatigue (Table 4).

**Table 4:** Humanistic outcomes related to fatigue in RRMS, adjusted results

	**β**	**SE**	**Exp(β)**	**95% CI**		***p* Value**
				**LCL**	**UCL**	
**SF-36v2**						
MCS Score^a^	−1.157	0.088	NA	−1.330	−0.983	<0.001
PCS Score^a^	−1.262	0.071	NA	−1.401	−1.123	<0.001
SF-6D Utility Score^a^	−0.017	0.001	NA	−0.018	−0.015	<0.001
**EQ-5D-5L**						
EQ-5D-5L Index Score^a^	−0.016	0.001	NA	−0.019	−0.013	<0.001
EQ-5D-5L VAS Score^a^	−2.263	0.194	NA	−2.643	−1.884	<0.001
**Depression**						
PHQ-9 Score^a^	0.117	0.008	1.124	0.101	0.133	<0.001
Moderate to Severe Depression Symptoms^b^	0.270	0.031	1.309	1.233	1.391	<0.001
**Anxiety**						
GAD-7 Score^a^	0.114	0.011	1.121	0.092	0.136	<0.001
Moderate to Severe Anxiety Symptoms^b^	0.227	0.034	1.255	1.173	1.342	<0.001
**PDQ-5 Score**^a^	0.726	0.030	NA	0.666	0.785	<0.001
**Overall Treatment Satisfaction**^**a,c**^	−0.068	0.0197	NA	−0.106	−0.029	0.001

For the set of tests shown in the table, the corrected α level was 0.050; all results remained statistically significant

*β* parameter estimate, *CCI* Charlson comorbidity index, *CI* confidence interval, *EQ-5D-5L* Euroqol-5 Dimensions-5 Levels, *Exp* exponential, *GAD-7* Generalized Anxiety Disorder-7, *LCL* lower confidence limit, *MCS* Mental Component Summary, *NA* not applicable, *PCS* Physical Component Summary, *PDQ-5* Perceived Deficits Questionnaire-5, *PHQ-9* Patient Health Questionnaire-9, *RRMS* relapsing-remitting multiple sclerosis, *SE* standard error, *SF-36v2* 36-Item Short Form Health Survey (version 2), *SF-6D* Short Form-6 Dimensions, *UCL* upper confidence limit, *VAS* visual analogue scale

^a^Generalized linear models controlling for age, race, sex, and CCI score; *N=*498

^b^Binary logistic regression model, controlling for age, sex, race, CCI score; *N=*498

^c^*n=*175 treated

In binary logistic regression models, moderate-to-severe symptoms for depression and anxiety were 1.31 and 1.26 times, respectively, more likely with higher fatigue score (Table 4). Additionally, moderate-to-severe symptoms for depression and anxiety were 10.45 (95% confidence interval [CI]: 6.38, 17.13; *p<*0.001) and 6.47 (95% CI: 3.75, 11.14; *p<*0.001) times, respectively, more likely in RRMS+HF than RRMS+LF (data not shown).

## Discussion

Fatigue disproportionately affects QoL in patients with MS [12–14], resulting in poor physical and mental health [14], as well as greater risk for developing depression [15]. Furthermore, fatigue in patients with RRMS has been implicated in disease progression [16] and work impairment [18]. Garg et al [37] reported greater functional disability, poor physical and mental HRQoL, and depression with higher fatigue; however, to our knowledge, there are no existing data on the burden by levels of fatigue, compared with the general population, along with the incremental burden of fatigue on humanistic outcomes in RRMS. This retrospective cross-sectional study aimed to fill this gap in the literature.

Previous studies have identified several sociodemographic- and disease-specific factors hypothesized to be associated with diminished HRQoL, including level of education, age, type of employment, physical activity, depression, disability level, type of MS, and social support [12, 13, 38–40]. Patients with MS have described fatigue as “time consuming and all-absorbing,” affecting daily functioning and social activities, lowering self-worth and cognitive ability, and increasing psychological distress, feelings of worthlessness and helplessness due to physical or emotional dependence, and negative feelings, such as despair, sadness, and sorrow [41]. Although studies have used different measures, fatigue has been repeatedly implicated as one of the factors linked to reduced HRQoL in patients with MS [14, 18, 37, 42–44].

Compared with matched non-MS controls and RRMS+LF in the present study, patients in the RRMS+HF cohort were less likely to be employed full time and reported lower exercise activity, both of which have been shown to be associated with HRQoL in patients with MS [12, 13]. Additionally, mean PCS scores were significantly lower in the RRMS+LF (42.31) and RRMS+HF (31.97) cohorts, compared with matched non-MS controls (49.56), representing an incremental difference of 7.25 and 17.59 points, respectively, thereby exceeding the minimally important difference (MID) of 3 points [45]. Similarly, the MIDs of 0.041 and 0.074 points were exceeded for mean SF-6D health utility scores (matched non-MS controls: 0.72; RRMS+HF: 0.53; RRMS+LF: 0.67) and EQ-5D-5L index scores (matched non-MS controls: 0.82; RRMS+HF: 0.58; RRMS+LF: 0.75) [46], respectively, indicating poorer HRQoL among patients with RRMS than matched non-MS controls, as well as greater humanistic burden in RRMS+HF than RRMS+LF. These findings demonstrate the importance of health care providers monitoring patient fatigue levels as a part of routine MS care to assess improvement versus an increase in severity, as fatigue level may be a general proxy for other important patient-centered outcomes and can inform provider recommendations for appropriate treatment.

Furthermore, fatigue score was found to be a predictor of poorer HRQoL, with significantly lower scores for PCS, MCS, SF-6D, and EQ-5D-5L, lower ratings of treatment satisfaction, and greater severity of depression, anxiety, and cognitive impairment after adjusting for covariates. Likewise, previous studies using the Multiple Sclerosis Quality of Life-54 (MSQoL-54) identified fatigue as an independent predictor of HRQoL in patients with MS [14, 47, 48]. In a previous study using the Fatigue Severity Scale and MSQoL-54 instrument, depression was assessed as a mediator between the relationship of fatigue and QoL, and it was estimated that the indirect effect mediated by depression accounted for 53.0% of the relationship [49]. Given this, future studies are warranted to examine the relationship between fatigue burden and QoL stratified by treatment type. Additionally, the mean PCS scores of patients with depression (40.14) [50] were comparable to RRMS+LF (42.31), but the scores of RRMS+HF were much lower (31.97). Mean PCS (46.61 vs 31.97), SF-6D health utility (0.62 vs 0.53), and EQ-5D index (0.71 vs 0.58) scores of patients with migraine were substantially higher than those reported by RRMS+HF [51], further emphasizing the burden of high fatigue in RRMS. Notably, in the aforementioned informal comparisons on mean PCS, SF-6D, and EQ-5D index scores between the RRMS+HF cohort from the current study and other serious health conditions from prior literature, all differences exceeded the MIDs for these measures, which suggests that the negative impact of high fatigue on HRQoL in MS may be clinically meaningful.

In the present study, patients with RRMS+HF were more likely to have severe depression and severe anxiety than RRMS+LF. This finding is consistent with a study by Greeke et al [15] in which individuals with MS having a higher level of fatigue exhibited greater risk for depression in addition to reduced physical and mental HRQoL, compared with individuals with low fatigue. Similarly, in a study by Chang et al [52], a strong correlation was observed between subjective fatigue and depression in patients with RRMS. Additionally, it was hypothesized in Chang et al [52] and in a review by Lee and Giuliani [53], that inflammation and cytokine production in response to MS may be associated with fatigue and depression in patients with MS, although results have been mixed across studies. Although the association is still not fully understood, there is agreement on the correlation between the immune system and depression and fatigue. In patients with MS, the correlation is less clear due to overlapping symptoms and the difficulty determining if symptoms are caused by the disease itself or because of the effects of treatment [53].

A recently published study indicated that perception of physical health can influence satisfaction and thus HRQoL in patients with MS [39]. Additionally, in the current study, treatment satisfaction was significantly lower in RRMS+HF than RRMS+LF, and RRMS+HF were more likely to report switching medications due to side-effects. In a prior prospective, cross-sectional, multicenter, observational study on therapy satisfaction in patients with RRMS (THEPA-MS), the authors reported convenience and fewer side effects as main factors associated with higher adherence [54]. Further analysis of the THEPA-MS study data revealed efficacy and side effects of treatment as independent predictors of physical and mental HRQoL in patients with RRMS [55]. Thus, limiting treatment side effects, such as MS-related fatigue, may potentially improve adherence and treatment satisfaction, which may have additional benefits regarding patients’ HRQoL.

Finally, MS not only imposes burden to patients and healthcare systems but also likely affects caregivers. Caring for patients with MS can negatively affect caregivers physically, psychologically, professionally, financially, and socially [56]. Furthermore, caregivers or spouses are at a higher risk for developing anxiety, depression, and lower QoL due to perceived burden of MS [57]. As such, understanding the primary or secondary causes of fatigue in patients with MS is important for patients and caregivers alike; along with clinical management, patients may see improved outcomes with regular exercise, physical therapy, and/or pharmacological management [58, 59]. Thus, minimizing the daily burden of fatigue may significantly improve patients’ physical and mental health, which may benefit not only patients, but families and society as well.

The results of the present study establish the profound effect of level of fatigue on HRQoL among patients with RRMS and demonstrate that worse outcomes are proportional to the level of fatigue. Considering the complex and multifactorial nature of MS-related fatigue, a systematic approach involving early diagnosis may help to improve HRQoL of patients with MS.

### Limitations

Although the NHWS is a nationally representative panel-based survey of the US adult general population, recruitment is not designed to be representative of any specific disease subpopulations, such as those with RRMS. Also, respondents who are likely to participate in an online survey may be systematically different from those who decide not to participate. For example, elderly patients with severe comorbidities or those with restricted internet access may be less likely to participate in online surveys. Furthermore, because data on diagnoses and health characteristics were self-reported, findings could not be confirmed independently through physician report, medical claims, or other objective sources. Additionally, because propensity score matching was performed for measured variables (age, race, sex, and CCI score), the study sample groups may differ on unmeasured variables. Also, no causal relationship between MS-related fatigue and outcomes can be established due to the cross-sectional nature of the data. Finally, we conducted multiple tests comparing groups, which may inflate the Type I error rate; however, we also provide corrected alpha thresholds for determining statistical significance for each set of tests. Hypotheses generated from the results of the current exploratory study may need to be tested in future research studies.

## Conclusion

The results of this study emphasize the significant association of fatigue on HRQoL and mental health in patients with RRMS. We observed that the overall impact on physical and mental HRQoL, as well as severity of depression and anxiety symptoms, was greater with higher level of fatigue. Additionally, the burden of fatigue on study measures was greater in RRMS+HF compared with RRMS+LF. These findings suggest that minimizing fatigue and related symptoms may improve the physical and emotional well-being of patients. This, in turn, may potentially have downstream implications for reducing stress-related comorbidities, including hypertension, heart disease, and sleep conditions, as well as improving interpersonal relationships with family members/caregivers, coworkers, and friends.

## Data Availability

The datasets analyzed during the current study are not publicly available NHWS is a proprietary database but are available from the corresponding author on reasonable request.

## Abbreviations

CCI: Charlson comorbidity index
CHAMPUS: Civilian Health and Medical Program of the Uniformed Services
CI: confidence interval
EQ-5D-5L: Euroqol-5 Dimensions-5 Levels
GAD-7: Generalized Anxiety Disorder-7
GLM: Generalized linear model
HCRU: Healthcare resource utilization
HF: High fatigue
HRQoL: Health-related quality of life
LCL: Lower confidence limit
LF: Low fatigue
MCS: Mental Component Summary
MFIS-5: 5-item Modified Fatigue Impact Scale
MID: Minimally important difference
MS: Multiple sclerosis
MSQoL-54: Multiple Sclerosis Quality of Life-54
NA: Not applicable
NHWS: National Health and Wellness Survey
PCS: Physical Component Summary
PDQ-5: Perceived Deficits Questionnaire-5
PHQ-9: Patient Health Questionnaire-9
QoL: Quality of life
RRMS: Relapsing-remitting multiple sclerosis
SD: Standard deviation
SE: Standard error
SF-36v2: 36-Item Short Form Health Survey (version 2)
SF-6D: Short Form-6 Dimensions
UCL: Upper confidence limit
US: United States
VA: Veterans Affairs
VAS: Visual analogue scale
